# Downregulation of Brf1 Induces Liver Failure and Inhibits Hepatocellular Carcinoma Progression by Promoting Apoptosis

**DOI:** 10.7150/jca.97277

**Published:** 2024-09-03

**Authors:** Yaping Xu, Chundong Yu, Hongbin Zhang, Tao Wang, Yujian Liu, Lupeng Wu, Shuping Zhong, Zaifa Hong

**Affiliations:** 1 Key laboratory of functional and clinical translational medicine, Fujian Province University, Xiamen Medical College, Xiamen, Fujian Province,China;; 2 Department of Hepato-Biliary-Pancreatic and Vascular Surgery, The First Affiliated Hospital of Xiamen University, School of Medicine, Xiamen University, Xiamen 361003, Fujian Province, China.; 3 State Key Laboratory of Cellular Stress Biology, Innovation Center for Cell Biology, School of Life Sciences, Xiamen University, Xiamen 361102, Fujian Province, China.; 4 Endoscopy Center, The First Affiliated Hospital of Xiamen University, School of Medicine, Xiamen University, Xiamen 361003, Fujian Province, China.; 5 Department of General Surgery, Xinglin District of the First Affiliated Hospital of Xiamen University, School of Medicine, Xiamen University, Xiamen 361022, Fujian Province, China.; 6 Department of Medicine, Keck School of Medicine, University of Southern California, Los Angeles, CA 90033, USA.

**Keywords:** Brf1, hepatocellular carcinoma, metabolism, Bcl-2, apoptosis

## Abstract

The occurrence and development of hepatocellular carcinoma (HCC) are closely related to abnormal apoptosis. Brf1 is highly expressed in HCC and has clinical prognostic value. Here, attenuation of Brf1-induced apoptosis was found, and the related mechanism was explored. In the study, general bioinformatics data for Brf1 were obtained from The Human Protein Atlas (HPA). Analyses of the clinical prognostic value of Brf1 in HCC were performed with the Xiantao Academic web server using R software. The basic data were obtained from the GTEx database and TCGA database. Brf1 conditional knockout mice were obtained by repeated mating of C57BL/6 Brf1LoxP/LoxP and C57BL/6 NS5A-alb-Cre-ERT2 mice and verified by genotyping. Liver function measurements, hematoxylin and eosin staining (HE), and immunohistochemistry (IHC) were performed to explore the cause of mouse death after Brf1 knockout. The Brf1 knockdown HCC cell model was generated using lentiviral vector-based shRNA transduction. Cell proliferation assays, plate colony formation assays, anchorage-independent colony formation assays and mouse subcutaneous tumor models were used to evaluate the progression of HCC. Western blot (WB) analysis, flow cytometry, and TUNEL assays were used to detect apoptosis. DNA sequencing, transcriptomics, and proteomics analyses were carried out to explore the antiapoptotic mechanism of Brf1. We found that Brf1 was highly expressed in HCC and had clinical prognostic value. Brf1 knockout led to liver failure and hepatocyte apoptosis in mice. Downregulation of Brf1 slowed HCC cell proliferation, colony growth, and mouse subcutaneous tumor growth and increased the sensitivity of HCC cells to apoptosis induced by chemotherapy drugs. The expression of Brf1 was positively related to that of the apoptosis gene Bcl-2. The sequencing, transcriptomics and proteomics analyses consistently showed that energy metabolism played an important role in Brf1 function, that protein-protein interaction was the primary mode, and that organelles such as mitochondria were the main sites. In Conclusions, downregulation of Brf1 inhibits HCC development by inducing apoptosis. Energy metabolism plays an important role in Brf1 function. These results provide a scientific basis for combating HCC.

## 1. Introduction

The incidence and mortality of hepatocellular carcinoma (HCC) are both high worldwide 1, 2. Primary liver cancer was the sixth most frequently diagnosed cancer and the third leading cause of cancer death worldwide in 2020 3. Although great progress has been made in the experimental research and clinical treatment of HCC in recent years, the molecular mechanism of HCC development is still unclear.

Apoptosis is a biological phenomenon. Abnormal apoptosis can cause cancer. Many genes are involved in the regulation of apoptosis, including oncogenes and tumor suppressor genes. Among these genes, ICE, Apaf-1, Bcl-2, Fas/APO-1, c-myc, p53, ATM, c-jun, etc., are closely related to HCC 4-6. Bcl-2 prevents the release of cytochrome C from mitochondria and inhibits apoptosis. Many studies have shown that Bcl-2 is closely related to the development of HCC^7^. The TFIIIB complex, which includes the Brf1 transcription factor along with the TATA-binding protein and B double prime subunit, operates in concert to facilitate gene transcription. Specifically, Brf1 is indispensable for the process of guiding RNA polymerase III (Pol III) to the appropriate target genes. Moreover, BRF1's activity is a critical factor that can limit the rate of transcription carried out by Pol III^8^. It was reported that elevated Brf1 levels are linked to worse outcomes in liver, breast, and stomach cancers^9^-^11^. However, there is no study on the relationship between Brf1 and apoptosis or between Brf1 and Bcl-2 in the development of HCC.

On the basis of the high expression of Brf1 in HCC and its clinical prognostic value, mice with conditional hepatic knockout of Brf1 were used to explore the role of Brf1 in the occurrence and development of HCC. Robust apoptosis was observed in the liver, and the mice gradually developed liver failure. Similarly, downregulation of Brf1 inhibited HCC proliferation, colony growth and subcutaneous tumor growth in mice. Brf1 had a positive regulatory effect on Bcl-2 expression. To explore the antiapoptotic mechanism of Brf1, gene sequencing, transcriptomics, and proteomics analyses were performed. The results suggested that Brf1, in terms of biological processes (BPs), influenced mitochondrial energy metabolism and hindered apoptosis. In terms of molecular functions (MFs), protein-protein interaction was an important mechanism. In terms of cell components (CCs), the cell membrane and organelles were the main sites.

In summary, Brf1/bcl-2 signaling axis induces apoptosis and promotes the development of HCC. Energy metabolism plays an important role in this signaling axis. This study may provide experimental evidence for improving the treatment of HCC.

## 2. Materials and methods

### 2.1 General bioinformatics analysis of Brf1

Data for Brf1 expression in normal tissues (protein/RNA distribution, subcellular localization, etc.) and Brf1 expression in cancer tissues (Brf1 protein expression in cancer tissues/cells, boxplots of Brf1 RNA expression in patients) were obtained from The Human Protein Atlas (HPA, https://www.proteinatlas.org).

### 2.2 Differential expression of Brf1 and clinical prognostic value of Brf1 in HCC

Clinical analyses, including the relationships between Brf1 and Fas, c-myc, and Bcl2, were performed with data from the Xiantao Academic web server (https://www.xiantao.love) using R software (version 3.6.3). Data for 50 normal liver samples were obtained from the GTEx database (https://www.gtexportal.org/). Data from 424 HCC patient samples, namely, 50 paracancerous tissues and 374 tumor tissues, were obtained from The Cancer Genome Atlas (TCGA, https://cancergenome.nih.gov/). All expression data were normalized by log2 conversion.

### 2.3 Mouse genotyping

C57BL/6 Brf1^LoxP/LoxP^ (BEIJING BIOCOTYGENE CO., Beijing, China) and C57BL/6 NS5A-alb-Cre-ERT2 (Experimental Animal Center of the Liver Disease Center in University of Southern California, California, USA) mice were repeatedly mated to obtain male C57BL/6 NS5A-alb-Cre-ERT2-Brf1^LoxP/LoxP^ mice. The study protocols were approved by the Institutional Animal Care and Use Committee of the University of South California (California, USA). DNA was extracted from mouse tails using the phenol/chloroform extraction method. Genotyping was carried out using a TAKARA PCR kit (Takara Bio, Shiga, Japan) according to the manufacturer's instructions with an ABI Prism 7700 Sequence Detection System (ThermoFisher Scientific Inc, Shanghai, China). The primer sequences were as follows: Brf1-LoxP Forward 5'- GGA TGT CCC ATC CCT GCT GAT G -3'_._ Reverse 5'- CTG CCA AAA GTG GCT GGG ACT TA -3'_._ Cre Forward 5^,^ -ATG CTT CTG TCC GTT TGC CG-3^'^_._ Reverse 5^'^ -CCT GTT TTG CAC GTT CAC CG--3^'^_._ GAPDH Forward 5^'^ -TCC ACC ACC CTG TTG CTG-3^'^_._ Reverse 5^'^ -ACC ACA GTC CAT GCC ATC-3^'^_._ NS5A Forward 5'- GGA CGA TGA GGA TCG TCG G -3^'^_._ Reverse 5^'^ -TGG CTA GCC GAG GAG CTG G -3'_._

### 2.4 Mouse liver function measurements

Mouse peripheral blood was extracted after euthanasia. The serum was separated, and total protein (TP), albumin (ALB), total bilirubin (TB), alkaline phosphatase (ALP), alanine transaminase (ALT), and aspartate transaminase (AST) were quantified using a liver function test kit (Nanjing Jiancheng Bio, Nanjing, China) according to the manufacturer's instructions with a spectrophotometer.

### 2.5 Hematoxylin and eosin (HE) staining and immunohistochemistry (IHC)

HE: Paraffin tissue blocks were cut sliced into 4 μm sections, deparaffined with xylene and rehydrated. The paraffin sections were stained sequentially with hematoxylin and eosin. IHC: Paraffin sections were submerged in 0.01 M citrate antigen retrieval buffer (pH 6.0) and microwaved for antigen retrieval. They were treated with 0.3% H_2_O_2_ for 15 min to block endogenous peroxidase at the designated times and were then treated with normal goat serum for 30 min to reduce nonspecific binding. Then, the sections were incubated with a rabbit polyclonal anti-Brf1 antibody (1:200) (Bethyl Laboratories, Inc., West FM, Montgomery, TX, USA) overnight at 4 °C. Then, the tissue sections were sequentially incubated with ready-to-use HRP-immunoglobulin (Evision^TM^) for 30 min, and color was developed with 3,3'-diaminobenzidine (DAB) as a chromogenic substrate. Then, the sections were costained with hematoxylin to visualize nuclei.

***2.6 Cell culture and transfection.*** The human liver cancer cell lines HepG2 and Huh7 were obtained from ATCC and cultured in DMEM supplemented with 10% fetal bovine serum and 100 U/ml penicillin and streptomycin in carbon dioxide incubator (Labtrip HWJ-3-160, Shanghai, China) that kept 37 °C in an atmosphere of 5% CO_2_. The cell line HepG2 was authenticated using Short Tandem Repeat (STR) analysis. The cell growth media were obtained from Life Technologies Inc. Endogenous Brf1 expression in HepG2 and Huh7 cells was downregulated using lentiviral vector-based shRNA transduction. The human Brf1 shRNA target sequences were 5′-cgGCATCTACAAGGAACACAA-3′ (shBrf1-103188), 5′-gcTTGAACAAGTCCTGTCAAA-3′ (shBrf1-103189) and 5′-GGTTGCAGCCAGAATGCATGA-3′ (shBrf1-103190). The shRNA control (sh-Ctrl) sequence was 5′- TTCTCCGAACGTGTCACGT -3′. The lentiviruses expressing the Brf1 shRNA plasmid and control shRNA plasmid were obtained from GeneChem (GeneChem Co., Ltd., Shanghai). Freshly plated HepG2 and HuH7 cells were infected with the lentiviruses and screened for stable transduction with puromycin according to the instructions.

### 2.7 Western blot analysis

Cell lysates were collected using the Total Protein Extraction Kit (Keygen, Jiangsu, China). Supernatants were recovered by centrifugation at 13,000 rpm for 15 minutes at 4 °C. Protein concentrations were measured, and equal amounts of total protein were separated by SDS-PAGE (Catalog #: PH0333. Network Technology Co., Ltd. Jiangsu, China). Proteins were transferred onto PVDF membranes (Merck Millipore, New York, USA), and the membranes were blocked for 1 hour in TBST. Then, the membranes were incubated overnight at 4 °C with primary antibodies. The rabbit anti-Brf1 antibody (Catalog #: A301-228A) obtained from Bethyl Laboratories. The mouse monoclonal antibody against actin (catalog #: sc-58673) was obtained from Santa Cruz Biotechnology. Anti-rabbit IgG-HRP and anti-mouse IgG-HRP were obtained from Cell Signaling. Chemiluminescence reagents were obtained from Santa Cruz Biotechnology. After washing with TBST, the membranes were incubated with the corresponding peroxidase-conjugated secondary antibodies for 1 hour at room temperature. Specific bands were detected using enhanced chemiluminescence (ECL) reagent (Perkin Elmer Life Sciences, Massachusetts, USA) on autoradiographic film. The film was attached to the membrane for exposure to display the protein band after the film is interacting with the ECL luminescent solution. The ChemiDoc EQ system with Quantity One software (Bio-Rad) was used to determine the densities of the protein bands.

### 2.8 Cell proliferation assay

HepG2 cells were seeded in six-well plates in triplicate. The cells were treated with PBS as a control and oxaliplatin as indicated in the figures. The confluence of the cells on the 6th day was approximately 85%-90%. Cell morphology was analyzed by microscopy using a Nikon Eclipse TE300 and MetaMorph software (Cell and Tissue Imaging Core of University of Southern California Research Center for Liver Diseases, P30 DK048522). Cells were assayed for viability through Trypan blue staining and observation of cell morphology, detached with trypsin and then counted with a globulimeter.

### 2.9 Plate colony formation assay

For the colony formation assay, stably transduced cells were seeded in 6-well plates at a density of 500 cells/well and incubated in an atmosphere of 5% CO_2_ for 14 days. Cells were fixed with 4% paraformaldehyde, stained with 0.5% crystal violet (Sigma‒Aldrich, St. Louis, USA) and allowed to dry. Colonies larger than 100 μm in diameter were counted.

### 2.10 Anchorage-independent colony growth

Stably transduced cells (1 x 10^4^ cells/well in a 6-well plate) were suspended in 0.35% (w/v) agar in 10% FBS/DMEM/F12 over a bottom layer of medium with 0.5% (w/v) agar. Cells were fed fresh complete medium twice weekly. Colonies were counted 2 or more weeks after plating as previously described^12^.

### 2.11 Subcutaneous tumor model in mice

The study protocols were approved by the Institutional Animal Care and Use Committee of Xiamen University (Xiamen, China, Animal Ethics Approval No. XMULAC20210090), and the mice were housed under specific pathogen‑free conditions (an environment free of specific disease‑causing microbes and parasites, including hepatitis A and E viruses; certain nonpathogenic microbes and parasites, including yeasts, were allowed to exist). All efforts were made to minimize suffering. Twenty mice were evenly divided into two groups. Xenograft tumors were established in the hind legs of 4- to 5-week-old nude mice (BALB/c, nu/nu: Shanghai SLAC Laboratory Animal Co., Ltd., Shanghai, China) by subcutaneous injection of 2x10^6^ cells. After a total of 14-21 days, the subcutaneous tumors were removed and cut into 1-mm^3^ sections. The tissue sections were implanted subcutaneously in nude mice (BALB/c, SPF grade, 4-5 weeks of age). The mice were administered oxaliplatin (20 mg/kg/week) by intraperitoneal injection on the 4^th^, 11^th^, and 18^th^ days after transplantation. The mice were sacrificed on the 21^st^ day after transplantation. The tumor volumes were determined according to the following formula: A×B2 /2, where A is the largest diameter and B is the perpendicular diameter.

### 2.12 Flow cytometry

Flow cytometry (Beckman Coulter Life Sciences, CA, USA) was used to examine apoptosis with an Annexin V-FITC/PI Apoptosis Detection Kit (Qcbio Science & Technologies Co. Ltd., Shanghai China) according to the manufacturer^'^s instructions.

### 2.13 TUNEL assay

Cell and tissue apoptosis were detected using a YF^R^488 TUNEL Assay Apoptosis Detection Kit (US EVERBRIGHT INC., Suzhou, China) according to the manufacturer^'^s instructions.

### 2.14 DNA sequencing

Brf1-binding DNA was obtained with a Hyperactive Universal CUT&Tag Assay Kit for Illumina (Vazyme Biotech Co., Ltd., Nanjing, China) following the manufacturer's instructions. The rabbit anti-human Brf1 antibody was obtained from Bethyl Laboratories, and goat anti-rabbit IgG was obtained from Vazyme (Vazyme Biotech Co., Ltd., Nanjing, China). CUT&Tag is a new method used to study protein-DNA interactions. Hyperactive pG-Tn5/pA-Tn5 Transposase, a new type of fusion enzyme with extremely high activity, precisely targets a DNA sequence near the gene encoding the target protein under the guidance of an antibody. Novogene Co., Ltd., was entrusted to complete the gene sequencing (Item No: X101SC22080089-Z01-J001-B1-7). The methods used are described in the supplemental material.

### 2.15 Transcriptomics and proteomics analyses

Applied Protein Technology Biotechnology Co., Ltd. (Shanghai, China), was entrusted to complete the transcriptomics and proteomics analyses (Transcriptomics Item No: P20211205614 and Proteomics Item No: P20211205612). The methods used are described in the supplemental material.

### 2.16 Statistical analysis

SPSS 16.0 statistical software (SPSS Inc., Chicago, IL, United States) was used for statistical analysis. All quantitative results are expressed as the means ± SDs. Statistically significant differences were identified using Student's t test or one-way ANOVA. The presented values are from three independent experiments. A value of P < 0.05 was considered to indicate a statistically significant difference. For the bioinformatics analysis, statistical data acquired from TCGA were merged and analyzed by R software (version 3.6.3).

## 3. Results

### 3.1 Brf1 was highly expressed in HCC and had clinical prognostic value

**Figure 1:**
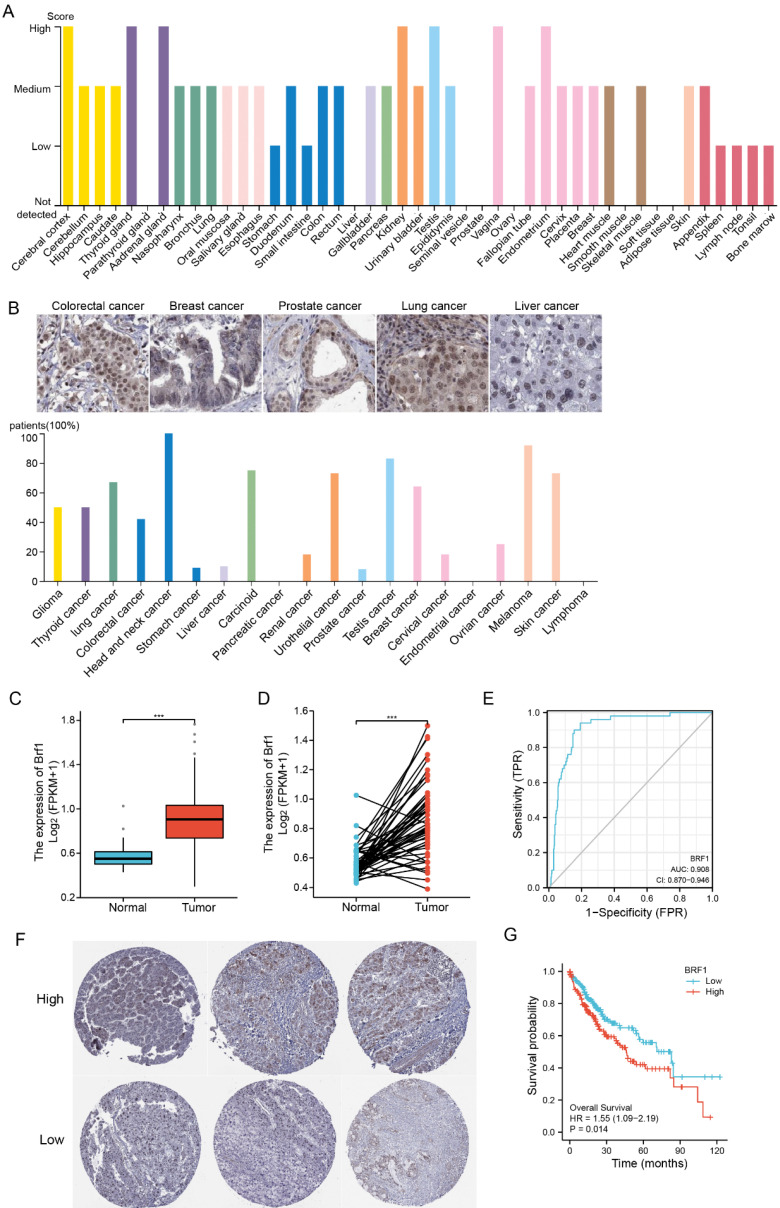
** Elevated Brf1 Expression in Hepatocellular Carcinoma (HCC) Correlates with Poor Prognosis.** (A) Tissue Distribution of Brf1. Brf1 is broadly expressed across various tissues but exhibits low levels in the liver. (B) Brf1 Expression in Cancer. High Brf1 expression is observed in a range of human tumor types according to the Human Protein Atlas (HPA). (C-D) Comparative Brf1 Expression in HCC. Statistically significant higher Brf1 levels in HCC tissues compared to normal liver tissues are demonstrated through paired (t-test, ***p < 0.0001) and unpaired (Wilcoxon rank-sum test, ***p < 0.0001) comparisons. (E) Receiver Operating Characteristic (ROC) Analysis. The area under the curve (AUC) of 0.908 with a confidence interval (CI) of 0.870-0.946 suggests that Brf1 is a highly specific and sensitive biomarker for HCC prognosis. (F)Variability of Brf1 Expression in HCC Tissues. Heterogeneous Brf1 expression patterns are evident in HCC tissues as depicted by the HPA.(G) Kaplan-Meier (K-M) Survival Curve. The log-rank test reveals a statistically significant difference in survival times between groups (*p = 0.029 < 0.05). Additionally, Cox regression analysis confirms a significant association between Brf1 expression and survival outcomes [hazard ratio (HR) = 1.55, 95% CI (1.09-2.19), *P = 0.014 < 0.05].

The Pol III gene plays an important role in regulating normal physiological development and maintaining the stability of the internal environment. A specific transcription factor for the pol III gene, Brf1 was found to be highly expressed in the nervous system, endocrine system, and urinary system but expressed at low levels in the liver (Fig. 1A). Brf1 expression had low specificity, including tissue specificity, cell type specificity, organ specificity, and cancer specificity. In human HCC tissue, Brf1 was mainly expressed in the nucleus^13^ (Table 1).

Brf1 was highly expressed in tissues of various human tumors (Fig. 1B). Its expression in tumor tissue was significantly higher than that in normal tissue (Fig. S1A). In HCC patients, in both unpaired comparisons groups (Fig. 1C) and paired comparisons (Fig. 1D), Brf1 expression in HCC tissue was significantly higher than that in normal liver tissue. ROC analysis showed that Brf1 had high sensitivity and specificity for diagnosing HCC (Fig. 1E). Brf1 expression varied among HCC tissues (Fig. 1F). Patients were divided into a high expression group and a low expression group based on the median Brf1 mRNA expression level (Table 2). The prognosis in the high Brf1 expression group was worse than that in the low Brf1 expression group. (Fig. 1G). Brf1 expression had clinical prognostic value for T stage (Fig. S1B), pathologic stage (Fig. S1C), AFP level (Fig. S1D, Table 2b), OS (Fig. S1E), Ishak fibrosis score (Table 2) and histologic grade (Fig. S1F) but not for N stage, M stage, pathologic grade, sex, race, age, weight, height, BMI, residual tumor status, adjacent hepatic tissue inflammation status, albumin level, prothrombin time, Child-Pugh score, Ishak fibrosis score, vascular invasion status, DSS, or PFI (Table 2). Due to differences in the data distribution and homogeneity of variance, some statistical data were corrected. For example, as the data exhibited a nonnormal distribution and inhomogeneity of variance, Welch's one-way ANOVA and Tukey's HSD post hoc test (for multiple hypothesis testing) were used to determine statistical significance for histologic grade (Tables 3, 4). These results indicated that HCC patients with high Brf1 expression had worse prognoses.

### 3.2 Brf1 knockout led to liver failure and hepatocyte apoptosis in mice

**Figure 2:**
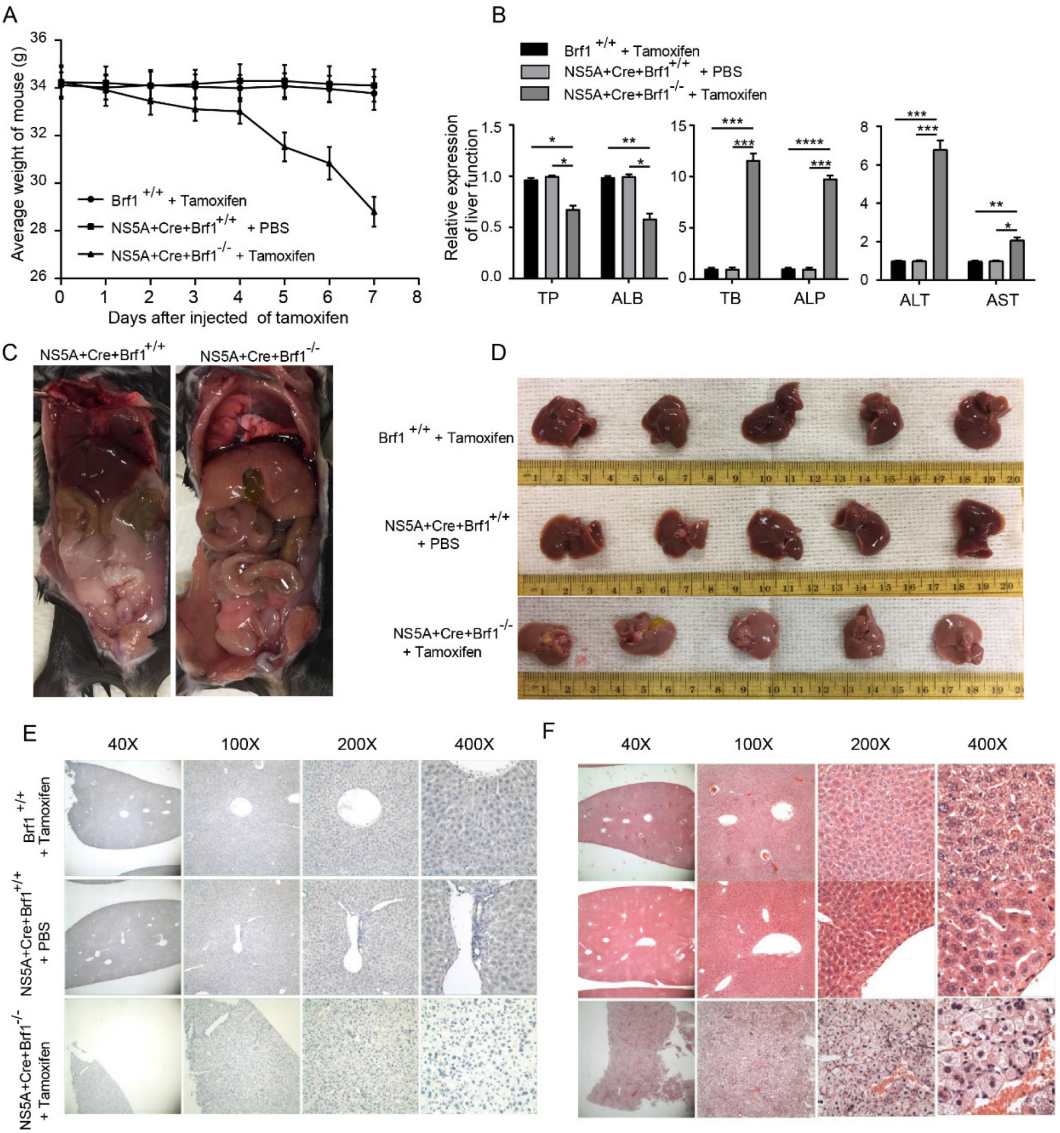
**Brf1 Deletion Results in Hepatic Dysfunction and Apoptosis in a Mouse Model**. (A) Mouse Weight Changes. Following tamoxifen injection, a progressive decrease in mouse weight was observed over the 7-day period, with statistical significance noted (t test, **p < 0.01). (B) Liver Function Markers. In Brf1 conditional knockout mice, levels of total protein (TP) and albumin (ALB) were reduced, while those of total bilirubin (TB) and alkaline phosphatase (ALP) were elevated. Additionally, alanine transaminase (ALT) and aspartate transaminase (AST) showed significant increases, indicative of liver injury (t test, **p < 0.01). (C) Gross Pathological Observations. Brf1 conditional knockout mice exhibited edema in digestive organs, with livers appearing swollen and pale. A notable accumulation of ascites was observed in the abdominal cavity. (D) Liver Morphology. Livers from Brf1 knockout mice were characterized by pallor and enlargement. (E) Verification of Brf1 Knockout. Brf1 expression was absent in the livers of knockout mice, contrasting with the weak expression observed in control groups. (F) Hepatocyte Pathology. The hepatic lobule structure in Brf1 knockout mice was severely disrupted, with disorganized hepatic plates and hepatocytes showing signs of swelling and apoptosis, evidenced by the presence of numerous apoptotic bodies.

Brf1 overexpression is important in the occurrence and development of HCC. A strategy for conditional (temporal dimension) knockout of Brf1 in the mouse liver (spatial dimension) with the NS5A gene was designed to study the role of Brf1 in HCC occurrence (Fig. S1G). The appetite and weight of the mice decreased gradually after Brf1 knockout (Fig. 2A). On the 7^th^ day, the mice were unable to drink. Their sclerae were clearly yellow, and the mice ultimately died (Fig. S1H). Liver function was tested to determine the cause of death. The synthesis of total protein (TP) and the albumin (ALB) level were decreased, the total bilirubin (TB) and alkaline phosphatase (ALP) levels were increased, and the alanine aminotransferase (ALT) and aspartate aminotransferase (AST) levels were significantly increased (Fig. 2B). The liver function time curve was plotted, and it showed that liver function gradually decreased (Fig. S1I). It did not change suddenly at a particular time point. We speculated that Brf1 gene expression was completely knocked out in mice 24 hours after intraperitoneal injection of tamoxifen. However, the Brf1 mRNA and Brf1 protein that were produced prior to injection could still function until they were completely degraded *in vivo*.

To understand how Brf1 affects liver function, the livers were removed and observed under a microscope after euthanasia. A large volume of ascites (about 5% of body weight) was found in the abdominal cavity in Brf1 conditional knockout mice, and the livers became pale and swollen (Fig. 2C, Fig. 2D). Brf1 was not expressed in mouse livers with Brf1 knockout and was weakly expressed in both control groups (Fig. 2E). Pathological analysis showed that the hepatic lobe structure was disorganized and that the structural integrity was destroyed. The arrangement of the hepatic plates was irregular.

The hepatocytes exhibited an increased volume of cytoplasm and were swollen, with a large number of apoptotic bodies (Fig. 2F). The pathology time curve was plotted based on the liver function time curve. There was no specific time point at which liver histology suddenly changed in Brf1 conditional knockout mice. With knockout of Brf1, hepatocytes gradually underwent apoptosis and lysis (Fig. S1J). In conclusion, Brf1 plays an important role in maintaining normal hepatocyte homeostasis and liver function 14. Brf1 knockout induces apoptosis in hepatocytes.

### 3.3 Downregulation of Brf1 slowed HCC progression and increased the sensitivity of HCC cells to apoptosis induced by chemotherapy drugs

**Figure 3:**
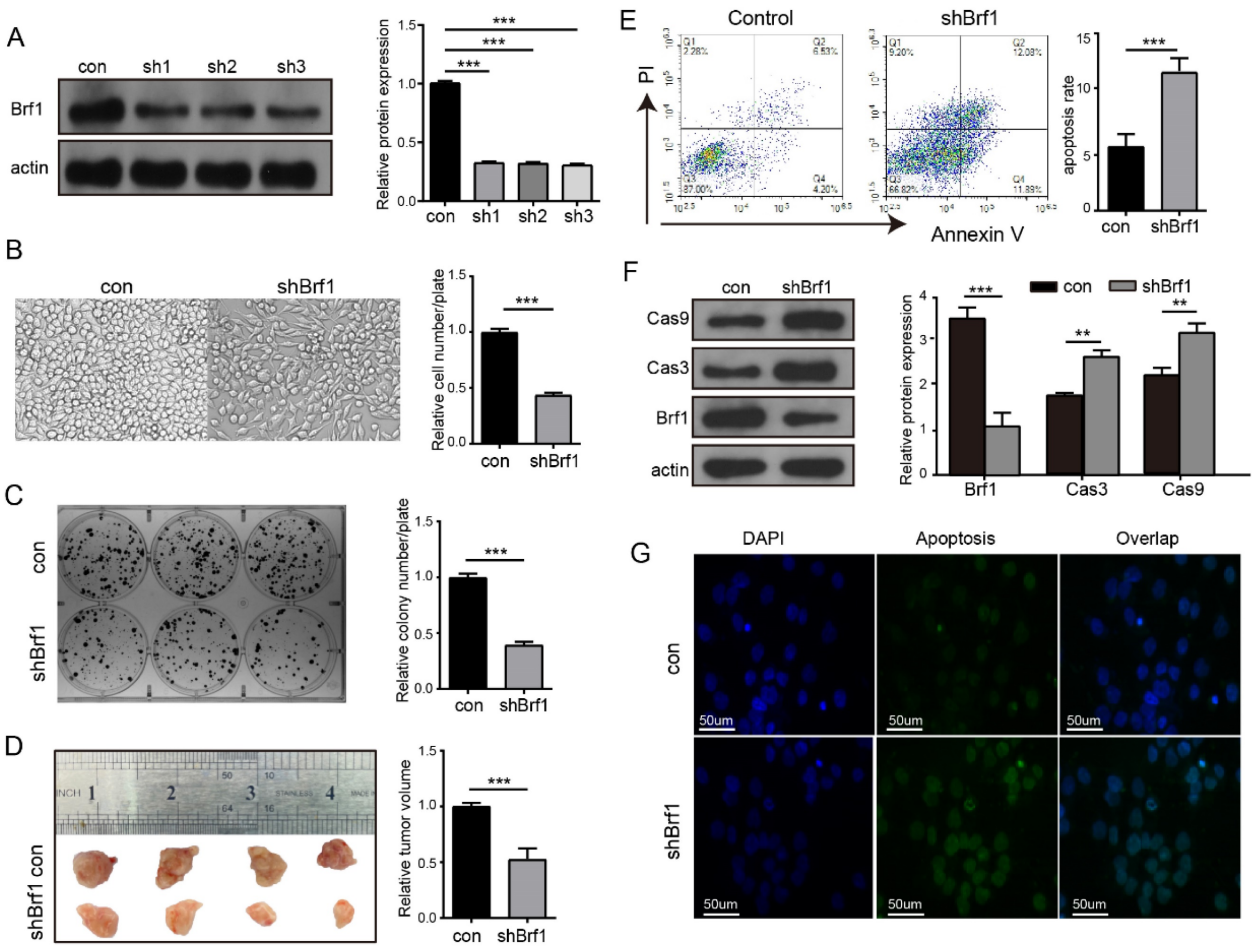
** Brf1 Suppression Attenuates Hepatocellular Carcinoma (HCC) Progression and Enhances Chemotherapy-Induced Apoptosis. (**A) Brf1 Knockdown Efficiency. Brf1 expression was significantly reduced by over 75% in sh1, sh2, and sh3 cells, as confirmed by the chi square test (***p < 0.001). (B-C) HepG2 Cell Proliferation and Colony Formation. Both the rate of proliferation and the ability to form colonies were markedly diminished in the Brf1 knockdown group, as determined by the t test (***p < 0.001). (D) Subcutaneous Tumor Growth in HepG2 Cells. Tumors developed more slowly in mice with Brf1 knockdown, indicating a retardation in tumor growth kinetics (t test, ***p < 0.001). (E) Apoptosis Assessment by Flow Cytometry. HepG2 cells were exposed to 400 µg/ml oxaliplatin for 24 hours to induce apoptosis. The Brf1 knockdown group exhibited a significantly higher rate of apoptosis compared to the control group (t test, ***p < 0.01). (F) Caspase 3 and Caspase 9 Expression. Expression levels of both caspase 3 and caspase 9 were elevated in the Brf1 knockdown group, suggesting enhanced apoptotic signaling (chi square test, **p < 0.01, ***p < 0.01). (G) TUNEL Assay for Apoptosis Detection. The sensitivity to oxaliplatin-induced apoptosis was increased in the Brf1 knockdown group, as evidenced by a higher number of TUNEL-positive cells.

A cell model of Brf1 knockdown was established to explore its role in HCC (Fig. 3A). HepG2 cell proliferation (Fig. 3B), plate colony formation (Fig. 3C) and agarose colony formation (Fig. S2A) were slowed in the Brf1 knockdown group. To verify the repeatability of these results in an animal experiment, HepG2 and Huh7 subcutaneous tumors were generated in mice. Tumor growth was also slower in the Brf1 knockdown group (Fig. 3D, Fig. S2B). Dose curves and time curves were generated to better understand the effect of oxaliplatin on HCC cells (Fig. S2C, S2D). Downregulation of Brf1 increased the sensitivity of HCC cells to apoptosis induced by oxaliplatin (Fig. 3E). Cas 3 and Cas 9 are important markers of apoptosis. Their expression levels in the Brf1 knockdown group were higher than those in the control group (Fig. 3F). The TUNEL assay results were consistent with the flow cytometry results (Fig. 3G). In the animal experiment, the mice were also treated with oxaliplatin. Apoptosis was more robust in tumor tissue from the Brf1 knockdown group (Fig. S2E). The decrease in Brf1 expression and the increase in apoptosis slowed the development of HCC.

### 3.4 Brf1 expression was positively related to Bcl-2 expression

**Figure 4:**
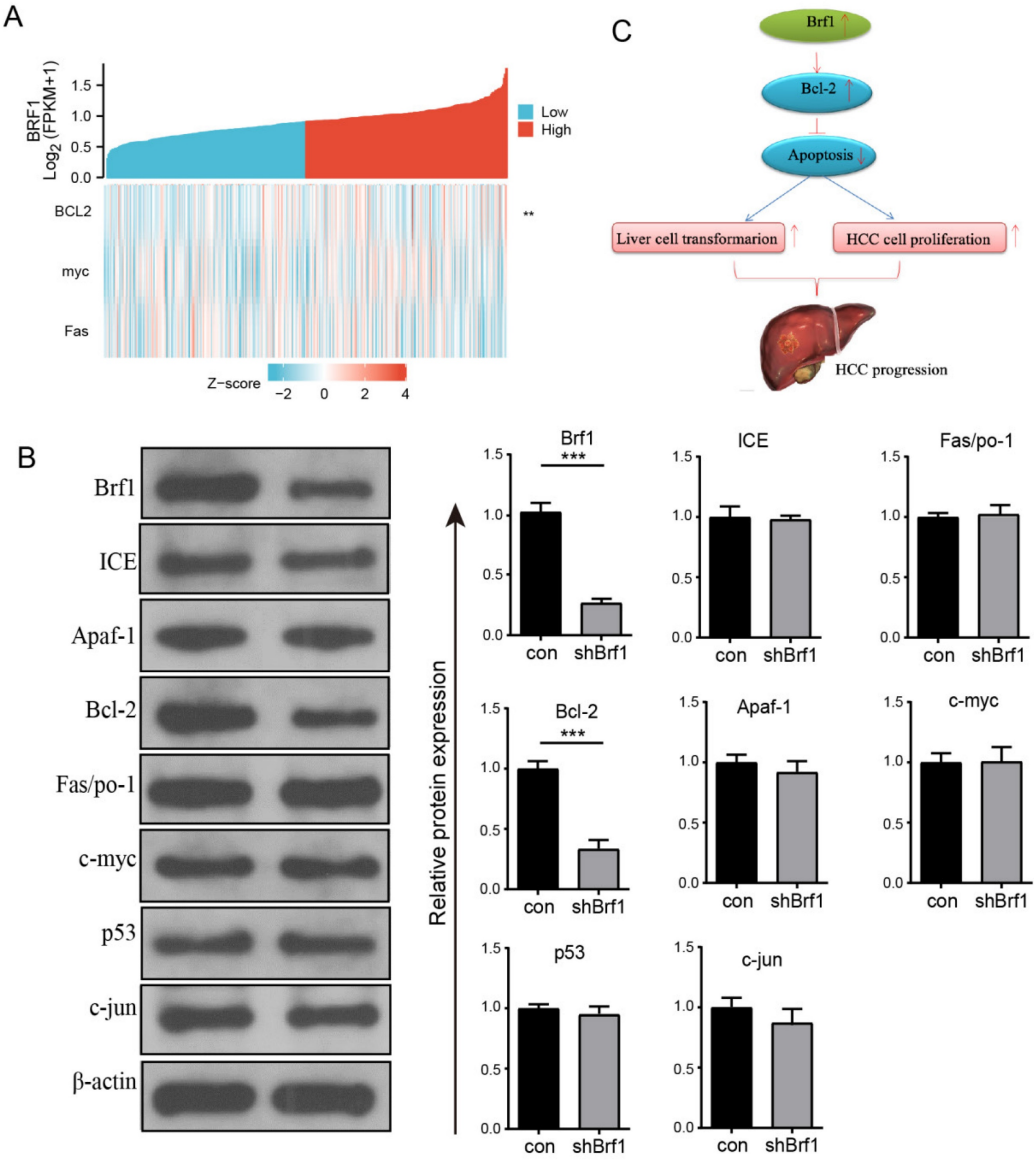
Positive Correlation Between Brf1 and Bcl-2 Expression and Its Implication in Apoptosis Inhibition and HCC Progression. (A) Single Gene Coexpression Heatmap. Bioinformatics analysis revealed a significant positive correlation between Brf1 and Bcl-2 expression, with no correlation observed for c-myc or Fas, suggesting a specific interaction between Brf1 and Bcl-2 in the context of apoptosis (not shown). (B) Schematic Illustration of Brf1's Role in Apoptosis and HCC Progression. The proposed model illustrates how Brf1 may upregulate Bcl-2 expression, thereby inhibiting apoptosis, promoting hepatocyte transformation, and accelerating HCC cell proliferation, ultimately leading to HCC progression. (C) Western Blot (WB) Validation of Apoptosis-Related Genes. WB analysis confirmed a positive relationship between Brf1 and Bcl-2 expression (***P < 0.001), while no significant association was found with ICE, Apaf-1, Fas/Apo-1, c-myc, p53, or c-jun.

The regulation of apoptosis involves many genes, including oncogenes and tumor suppressor genes. Among these genes, ICE, Apaf-1, Bcl-2, Fas/Apo-1, c-myc, p53, ATM, and c-jun are closely related to HCC. The above genes were investigated individually. The basic clinical data for Brf1 and ICE, Apaf-1, Bcl-2, Fas/Apo-1, c-myc, p53, and c-jun from TCGA were analyzed on the Xiantao Academic website. Brf1 expression was positively correlated with Bcl-2 expression (Fig. 4A, Table 5). The WB results further confirmed that the expression of only Bcl-2 was positively correlated with that of Brf1 (Fig. 4B). Bcl-2, a membrane integrin, inhibits the release of cytochrome C from mitochondria during apoptosis. An increasing number of studies have indicated that Bcl-2 is closely related to the development of HCC. Pyrimidine inhibits the apoptosis of HCC cells by upregulating Bcl-2. miR-365 induces HCC cell apoptosis by targeting Bcl-2 5, 15. Our results suggest that in the microenvironment of liver cancer, an increase in Brf1 may promote the expression of Bcl-2, thereby promoting the proliferation of liver cancer cells and inhibiting their apoptosis. (Fig. 4C).

### 3.5 Energy metabolism played an important role in Brf1 function

The antiapoptotic mechanism of Brf1 was explored by bioinformatics approaches. Energy metabolism plays an important role in Brf1 function. Protein-protein interaction was the primary mode. Intracellular organelles, such as mitochondria, were the main sites.

**Figure 5:**
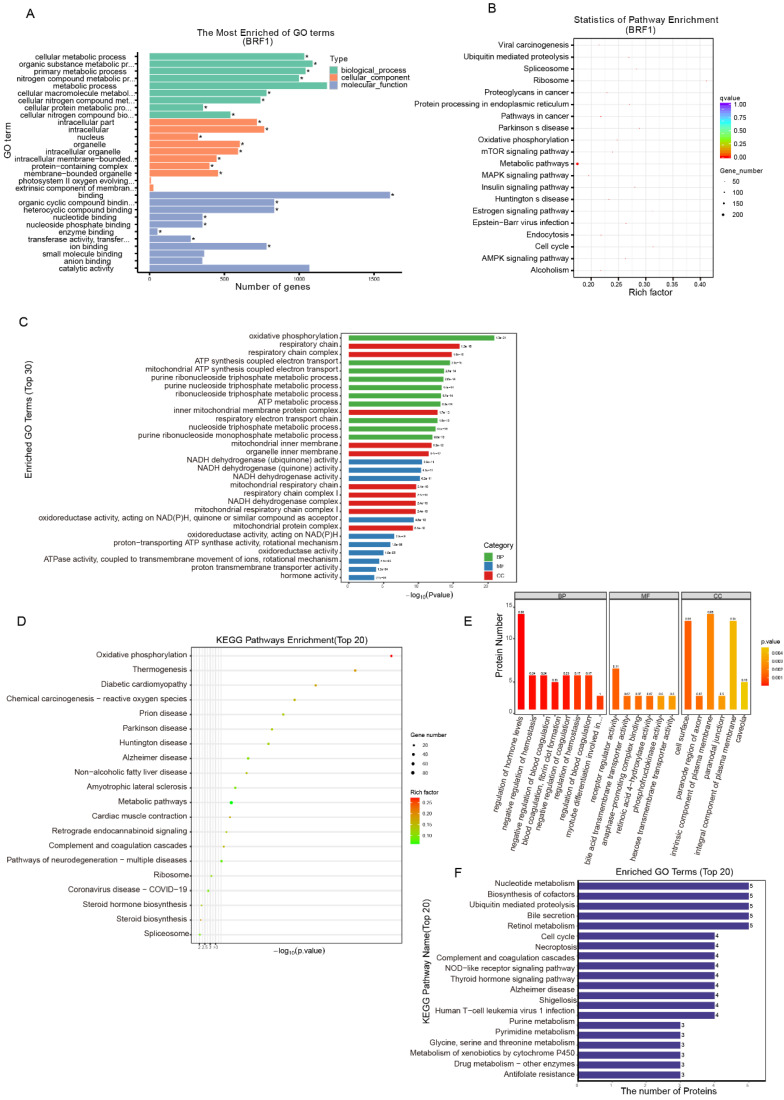
** Brf1's Integral Role in Energy Metabolism and Its Association with Metabolic Pathways**. (A) Sequencing-GO Enrichment Analysis Plot. Within the Biological Process (BP) category, Brf1 is linked to metabolic processes, particularly those involving cellular macromolecules. In the Cellular Component (CC) category, Brf1 shows an association with membranous organelles, including mitochondria. In the Molecular Function (MF) category, Brf1 is related to binding functions, such as heterocyclic compound binding. (B) Sequencing-KEGG Enrichment Scatter Plot. Brf1 demonstrates a significant connection to metabolic pathways, indicating its potential regulatory role in these processes. (C) Transcriptomic-GO Enrichment Histogram. In the lower panel, Brf1 is associated with terms related to energy metabolism, with a notable emphasis on mitochondrial respiration. (D) Transcriptomic-KEGG Enrichment Bubble Plot. In the lower panel, Brf1 is implicated in two major pathway categories: energy metabolism and chronic diseases. (E) Proteomic-GO Annotation Statistical Diagram of DEGs. Within the BP category, enrichment in the regulation of metabolism and blood coagulation indicates Brf1's significance in liver function. In the CC category, Brf1 is closely associated with the cell and mitochondrial membranes. In the MF category, enrichment in receptor regulator activity terms suggests that Brf1 may function through protein-protein interactions. (F) Proteomic-KEGG Pathway Annotation Statistical Diagram (Top 20). Brf1 is related to several metabolic pathways, including steroid hormone biosynthesis, nucleotide metabolism, and cell death/apoptosis pathways such as cell cycle, necroptosis, and metabolism of xenobiotics by cytochrome P450.

The Brf1 binding sequence was obtained by CUT&Tag. Brf1 is a transcription factor that binds to a promoter sequence near the transcription start site (TSS) to enhance gene transcription. The signal surrounding the TSS was significantly enhanced. Brf1 bound to a site approximately 300 bp upstream of the TSS (Fig. S3A). The lengths of the Brf1 binding sites were approximately 75 bp and 125 bp (Fig. S3B). Both the number of Brf1 binding sites and the fraction of reads in peaks (FRiP) were high, indicating that Brf1 bound to a wide range of sites and affected many downstream genes (peak number: 4000; FRiP: 3.4283%). The distribution map of the peaks in gene functional regions confirmed that Brf1 was also a promoter of transcription (Fig. S3C). Based on the GO annotations, Brf1 affects energy metabolism and participates in protein-protein interactions in intracellular organelles such as mitochondria (Fig. 5A, Table 6). Based on the KEGG annotations, Brf1 also affects cellular energy metabolism (Fig. 5B, Table 7). Brf1 may promote energy metabolism and enhance ribosome function, which results in accelerated cell cycle progression and decreased apoptosis, ultimately leading to the occurrence and development of tumors.

The general GO functional enrichment analysis results of the transcriptome data showed that in the BP category, Brf1 was mainly related to terms associated with nucleic acid metabolism and energy metabolism, especially mitochondrial respiration. In the MF and CC categories, Brf1 was determined to play a role through protein-protein interactions in various intracellular organelles, such as mitochondria and the endoplasmic reticulum (Fig. S3D). The GO enrichment histogram details the general GO enrichment patterns (lower panel) (Fig. 5C). Most of the downregulated genes were related to energy metabolism, especially mitochondrial respiration. They were also related to nucleic acid metabolism. KEGG enrichment analysis showed that Brf1 was mainly related to two kinds of pathways (Fig. 5D, Fig. S3E). One was energy metabolism pathways, such as oxidative photosynthesis. The other was the occurrence and development of chronic diseases, such as nonalcoholic fatty liver disease.

Proteomics analysis showed that Brf1 expression resulted in downregulation of 59 proteins and upregulation of 76 proteins (Table 8). Eight of the downregulated proteins and 10 of the upregulated proteins had good repeatability (Table 9). Based on the above information, Brf1 may exert antiapoptotic effects in the following ways. (1) Interference with the DNA repair ability in the nucleus and mitochondria. (2) Increasing the energy generated by glycolysis and mitochondrial respiration. (3) Enhancing the function of RNA polymerase III. Because these were the consistent differences across all samples, they were of great importance. GO functional enrichment analysis showed that in the BP category, Brf1 was mainly involved in the regulation of hormones related to energy metabolism, such as thyroid hormone and growth hormone (Fig. S3F). The domain enrichment analysis diagram indicates that Brf1 has a great impact on cellular energy metabolism and blood coagulation function (Fig. S3G). In the CC category, Brf1 was closely related to the cell membrane and organelle membrane and participated in the extrinsic apoptosis pathway or mitochondrial apoptosis pathway (Fig.S3H). In the MF category, the enrichment in receptor regulator activity terms suggests that Brf1 may play a role through protein-protein interactions. In addition, the enrichment in the bile acid transporter activity term also suggested that Brf1 was closely related to liver metabolic function (Fig. S3I). The general GO annotation statistical diagram further details the results of the above analysis (Fig. 5E). The top 20 KEGG pathway annotations showed that Brf1 was mainly involved in energy metabolism and cell apoptosis (Fig. 5F).

## 4. Discussion

Brf1 is a specific transcription factor for the Pol III gene and is expressed widely throughout the body. Generally, Pol III gene transcription is active, and the levels of its downstream products tRNA and 5S rRNA are high in tumor cells 10, 16, 17. Brf1 is highly expressed in various human tumors, including HCC. Various oncogenes and tumor suppressor gene products can regulate the transcriptional activity of Brf1 and thus affect the transcript level of Pol III 11. Professor Shuping Zhong reported that reducing the expression of Brf1 inhibited the transcription of the Pol III gene, limited the transformation of immortalized hepatocytes, and slowed the growth and clone formation of HCC cells and the growth of transplanted HCC tumors in nude mice 18-21. There are literature reports that the knockout of Brf1 leads to embryonic lethality at the blastocyst stage and that the conditional deletion of Brf1 in gastrointestinal epithelial tissues, intestines, liver, and pancreas is incompatible with organ homeostasis.

The deletion of Brf1 in the adult intestine and liver induces apoptosis. Brf1 plays a permissive rather than a driving role in the development of pancreatic and intestinal epithelial cancers^14^. Alcohol induces the expression of Brf1 in hepatocytes^10^ and may be related to the activation of JNK1^22^ Our results showed that Brf1 was significantly related to the AFP level, histological grade, OS, and Ishak fibrosis score. These results suggested that Brf1 was closely related to the development of HCC.

To study the role of Brf1 in the occurrence and development of HCC, a genetically modified mouse model was designed. Brf1 conditional knockout mice (NS5A-alb-Cre-ERT2-Brf1^loxp/loxp^) were distinguished by four aspects. First, NS5A is an oncogenic hepatitis C virus protein. Male mice expressing the NS5A gene fed a diet containing alcohol (the Lieber- DeCarli liquid diet, which contains an amount of alcohol roughly equal to 3 drinks/day) for 8 months had an HCC tumorigenesis rate of 100%. Second, the Cre gene was located downstream of the albumin gene. It was expressed only when the albumin gene was expressed. This strategy allowed us to determine spatially that Brf1 was knocked out only in the liver, while other organs were not affected. Third, Cre-ERT2 functioned only under activation by tamoxifen. In this way, Brf1 knockout was conditional in space and time. Fourth, we can use alcohol not only to naturally induce HCC tumorigenesis in mice to study the role of Brf1 in the occurrence of HCC but also to study the role of Brf1 in the development of HCC after tumor formation. However, liver function deteriorated greatly after Brf1 knockout. The liver is an important metabolic organ, and the mice did not survive longer than eight days. When Brf1 was knocked out, normal hepatocytes underwent apoptosis and died.

Based on the above findings, an interesting question emerged. Could knockout or knockdown of Brf1 also induce apoptosis in HCC cells and inhibit the development of HCC? Normal apoptosis plays an active role in ensuring cell growth and physiological development and maintaining the stability of the internal environment. Abnormal cell apoptosis promotes the occurrence and development of gastric cancer, HCC, colon cancer, and other malignant tumors 23. There have been many reports on the relationship between apoptosis and HCC development. Many molecules, such as miR-223-3, miRNA-1297, and BRD8, promote or inhibit the development of HCC by regulating apoptosis 24-26. Professor Xing Lin also noted that isoorientin in eupatorium induced apoptosis in HCC cells through the mitochondrial pathway and inhibited the development of HCC 27. The normal expression of the Brf1 and Pol III genes also plays an important role in maintaining physiological development and the stability of the internal environment. Dritan Liko *et al*. reported that loss of Brf1 function in the embryonic stage led to dysplasia of the liver, pancreas, and small intestine. Loss of Brf1 function led to homeostasis disorders of the liver, pancreas, and small intestine in mature mice 14. YH Jee *et al*. reported that mutations in the Brf1 gene caused bone and nervous system developmental disorders in children 14, 28, 29. Our experiments also showed that a large number of apoptotic bodies appeared in hepatocytes and that liver function worsened significantly in mice with conditional Brf1 knockout in the liver. There have been few reports on the relationship between Brf1 and apoptosis. Combining the evidence that Brf1 dysregulation promoted the development of HCC and that abnormal apoptosis promoted the development of HCC, we speculated that Brf1 promoted the development of HCC through inhibition of apoptosis. Can downregulation of Brf1 enhance apoptosis in HCC cells and then inhibit the development of HCC? Our results showed that downregulation of Brf1 inhibited HCC cell growth and clone formation, as well as subcutaneous tumor growth in mice. This was consistent with Dr. Lin's report that upregulation of Brf1 by alcohol administration promoted HCC cell growth and clone formation 30. In general, the rate of apoptosis in wild-type HepG2 and shBrf1 HepG2 cells was low. Brf1 knockdown increased the sensitivity of hepatoma cells to apoptosis induced by oxaliplatin. This finding provided an effective way to improve the chemosensitivity of HCC.

How does Brf1 regulate apoptosis? The relationships between the expression of Brf1 and the HCC apoptosis-related genes ICE, Apaf-1, Bcl-2, Fas/APO-1, c-myc, p53, and c-jun were explored by Western blotting. Brf1 expression was positively related only to Bcl-2 expression. Regression analysis of data from the TGCA database also suggested that Brf1 expression was positively related to Bcl-2 expression. Many studies have shown that Bcl-2 is closely related to HCC. TTP increased the sensitivity of head tumors to cisplatin by downregulating Bcl-2^31^. A novel pregnenolone derivative modulated apoptosis via Bcl-2 family genes in HCC 32. Follistatin-like protein 5 inhibited HCC progression by inducing caspase-dependent apoptosis and the expression of Bcl-2 family proteins 33. Abnormal expression of IL-32α in HCC inhibited cell growth and induced apoptosis through the inactivation of NF-κB and Bcl-2 34.

Sequencing, transcriptomics and proteomics approaches were used to further explore the mechanism by which Brf1 inhibits apoptosis. The results of these analyses consistently suggested that Brf1 was mainly related to energy metabolism, especially mitochondrial respiration and cell death/apoptosis, in terms of BPs. The organelle membrane was identified as the main site in the CC category. Protein-protein interaction was identified as the major mode in the MF category. We speculated that Brf1 promoted energy metabolism and mitochondrial function, which accelerated cell cycle progression, inhibited apoptosis, and ultimately led to the development of tumors.

The abnormal expression of Brf1 was related with the high level of phosphorylated AMPKα (pAMPKα)^35^. AMPK, a serine/threonine kinase, is activated under conditions of energy deficiency, enhancing ATP production and curtailing its consumption. Recent studies have shed light on an ancestral role of AMPK in safeguarding mitochondrial health, with numerous newly identified AMPK substrates implicated in various facets of mitochondrial homeostasis, including the process of mitophagy^36^. Energy metabolism is a basic function of cells. When metabolic disorders occur, the occurrence and development of tumors, including HCC, is promoted 37-39. Abnormal glucose metabolism promotes the proliferation of tumor cells 40. A large number of studies have shown that aerobic glycolysis is prevalent in a variety of cancers, including breast cancer^41^, gastric cancer 42 lung cancer 43 pancreatic cancer 44, colorectal cancer^45^. Bustamante E, Beyolu D 46 and Li S 47 demonstrated that inhibition of aerobic glycolysis in HCC cells promoted cell death. Many studies have confirmed that glycolysis is closely related to the development and drug resistance of HCC 48-50. Mitochondria are important sites of energy metabolism, and mitochondrial dysfunction is also closely related to chronic liver diseases as well as HCC 51. Various key enzymes in energy metabolism affect the cell cycle and affect the progression of HCC 52. However, there have been few reports on the direct relationship between energy metabolism and apoptosis, especially regarding the apoptosis-related molecule Bcl-2.

## 5. Conclusions

In this study, a large number of apoptotic bodies were found in Brf1 conditional knockout mice. Downregulation of Brf1 inhibited HCC development and increased the sensitivity of HCC cells to apoptosis induced by chemotherapy drugs. Brf1 expression was positively related to Bcl-2 expression. Energy metabolism played an important role, protein-protein interactions were a key mode, and the organelle membrane was the main site of this process. However, can full knockout of Brf1 induce complete HCC cell apoptosis and lead to HCC cell death? How does Brf1 regulate energy metabolism? How does energy metabolism regulate Bcl-2 expression or apoptosis? These issues need intensive exploration in the future.

## Supplementary Material

Supplementary figures.

## Figures and Tables

**Table 1:** Summary of Brf1.

Protein	BRF1, RNA polymerase Ill transcription initiation factor subunit
Gene name	BRF1 (BRF, GTF3B, hBRF, TAF3B2, TAF3C, TFIIIB90)
Tissue specificity	Low tissue specificity
Single cell type specificity!	Low cell type specificity
Blood specificity	Low immune cell specificity
Brain specificity!	Low human brain regional specificity
cancer prognostic summary	Prognostic marker in pancreatic cancer (favorable) and renal cancer (favorable).
Predicted location	Intracellular
Subcellular summary	Located in Nucleoplasm, Nuclear bodies (Single cell variability, CCD Protein)
Protein function (UniProt)	General activator of RNA polymerase which utilizes different TFIIIB complexes at structurally distinct promoters., The isoform 1 is involved in the transcription of tRNA, adenovirus VA1, 7SL and 5S RNA. Isoform 2 is required for transcription of the U6 promoter.
Molecular function (UniProt)	Activator
Biological process (UniProt)	Transcription, Transcription regulation
Disease involvement	Disease mutation, Dwarfism. Mental retardation
Ligand (UniProt)	Metal-binding, Zinc
Gene summary (Entrez)	This gene encodes one of the three subunits of the RNA polymerase Ill transcription factor complex. This complex plays a central role in transcription initiation by RNA polymerase Ill on genes encoding tRNA, 5S rRNA and other small structural RNAs. The gene product belongs to the TF2B family. Several alternatively spliced variants encoding different isoforms, that function at different promoters transcribed by RNA polymerase Ill, have been identified.

**Table 2:** a. Clinical Baseline Data 1; b. Clinical Baseline Data 2.

a. Clinical Baseline Data 1.				
Characteristics	Low expression of BRF1	High expression of BRF1	P value	Method
n	187	187		
# Pathologic T stage, n (%)			0.141162207	Chisq test
T1	100 (27%)	83 (22.4%)		
T2	47 (12.7%)	48 (12.9%)		
T3	32 (8.6%)	48 (12.9%)		
T4	5 (1.3%)	8 (2.2%)		
Pathologic N stage, n (%)			1	Yates' correction
N0	128 (49.6%)	126 (48.8%)		
N1	2 (0.8%)	2 (0.8%)		
Pathologic M stage, n (%)			0.614459893	Yates' correction
M0	133 (48.9%)	135 (49.6%)		
M1	3 (1.1%)	1 (0.4%)		
# Pathologic stage, n (%)			0.050099469	Yates' correction
Stage I	94 (26.9%)	79 (22.6%)		
Stage II	47 (13.4%)	40 (11.4%)		
Stage III	33 (9.4%)	52 (14.9%)		
Stage IV	4 (1.1%)	1 (0.3%)		
Tumor status, n (%)			0.518936422	Chisq test
Tumor free	106 (29.9%)	96 (27%)		
With tumor	75 (21.1%)	78 (22%)		
Gender, n (%)			0.740197323	Chisq test
Female	59 (15.8%)	62 (16.6%)		
Male	128 (34.2%)	125 (33.4%)		
Age, n (%)			0.718639414	Chisq test
<= 60	87 (23.3%)	90 (24.1%)		
> 60	100 (26.8%)	96 (25.7%)		
Histological type, n (%)			0.206609844	Yates' correction
Fibrolamellar carcinoma	3 (0.8%)	0 (0%)		
Hepatocellular carcinoma	181 (48.4%)	183 (48.9%)		
Hepatocholangiocarcinoma	3 (0.8%)	4 (1.1%)		
				
**b.** Clinical Baseline Data 2.				
Characteristics	Low expression of BRF1	High expression of BRF1	P value	Method
# Histologic grade, n (%)			0.032990246	Chisq test
G1	33 (8.9%)	22 (6%)		
G2	94 (25.5%)	84 (22.8%)		
G3	50 (13.6%)	74 (20.1%)		
G4	8 (2.2%)	4 (1.1%)		
# AFP (ng/ml), n (%)			0.012290126	Chisq test
<= 400	124 (44.3%)	91 (32.5%)		
> 400	26 (9.3%)	39 (13.9%)		
# Fibrosis Ishak score, n (%)			0.017729192	Yates' correction
0	46 (21.4%)	29 (13.5%)		
1/2	16 (7.4%)	15 (7%)		
3/4	8 (3.7%)	20 (9.3%)		
5	4 (1.9%)	5 (2.3%)		
6	46 (21.4%)	26 (12.1%)		
Vascular invasion, n (%)			0.513554307	Chisq test
No	112 (35.2%)	96 (30.2%)		
Yes	55 (17.3%)	55 (17.3%)		
Adjacent hepatic tissue inflammation, n (%)			0.525787246	Chisq test
None	66 (27.8%)	52 (21.9%)		
Mild	53 (22.4%)	48 (20.3%)		
Severe	12 (5.1%)	6 (2.5%)		
# OS event, n (%)			0.082324087	Chisq test
Alive	130 (34.8%)	114 (30.5%)		
Dead	57 (15.2%)	73 (19.5%)		
DSS event, n (%)			0.490115515	Chisq test
No	147 (40.2%)	140 (38.3%)		
Yes	37 (10.1%)	42 (11.5%)		
PFI event, n (%)			0.756314874	Chisq test
No	97 (25.9%)	94 (25.1%)		
Yes	90 (24.1%)	93 (24.9%)		

# Brf1 had clinical prognostic significance.

**Table 3:** Histologic grade relevance- Welch One-way Anova Test.

Group	(DFn)	(DFd)	statistics	*p* value
Intragroup comparison	3	47.139	3.0239	0.0387

**Table 4:** Histologic grade- multiple hypothesis test.

Group I	Group J	Estimated value(J-I)	(95%CI)	Adj. P value
G3	G2		-	NA
G3	G1		-	NA
G3	G4	-0.38048	-0.7484532	0.0446
G2	G1		-	NA
G2	G4	-0.29118	-0.738371	0.1769
G1	G4	-0.18556	-0.7888	0.6182

**Table 5:** Molecular correlation table.

Target mol.	Comparative mol.	Related coefficient (Pearson)	P value (Pearson)	Related coefficient (Spearman)	P value (Spearman)
BRF1	bcl2	0.181	< 0.001	0.139	0.007**
BRF1	myc	0.049	0.344	0.066	0.2
BRF1	fas	-0.104	0.045*	-0.092	0.075

**Table 6:** Sequencing-GO enrichment list.

GO	Description	Term type	Overrepresented p value	Corrected *p* value	Gene item	Gene list	Bg item	Bg list
GO:0044237	cellular metabolic process	Biological process	2.40E-08	5.76E-05	1034	2.36E+03	1.10E+04	28465
GO:0044424	intracellular part	Cellular component	8.20E-08	0.00013107	718	2.36E+03	7.92E+03	28465
GO:0097159	organic cyclic compound binding	Molecular function	1.58E-07	0.0001516	833	2.36E+03	8.17E+03	28465
GO:1901363	heterocyclic compound binding	Molecular function	1.58E-07	0.0001516	833	2.36E+03	8.17E+03	28465

**Table 7:** Sequencing-KEGG enrichment list.

Term	Database	ID	Input number	Background number	P value	Corrected *p* value
Ribosome	KEGG PATHWAY	hsa03010	57	138	4.42E-21	6.41E-19
Pathways in cancer	KEGG PATHWAY	hsa05200	87	397	3.53E-16	3.41E-14
Epstein-Barr virus infection	KEGG PATHWAY	hsa05169	54	204	2.80E-13	2.03E-11
Cell cycle	KEGG PATHWAY	hsa04110	39	124	7.40E-12	4.29E-10

**Table 8:** Proteins difference analysis table.

Filtering Criteria	Fold change > 2.0 and p value < 0.05		Protein which identified at least two of three replicates in one group while another group with all null value	
Comparisons	Significantly changing		Consistent presence/absence	
	Upregulated	Downregulated	Upregulated	Downregulated
Brf1 C vs Brf1 E	76	59	10	8

**Table 9:** Different expression Proteins with good repeatability table.

Protein	Protein Name	Gene Name
O43677	NADH dehydrogenase [ubiquinone] 1 subunit C1, mitochondrial	NDUFC1
P00740	Coagulation factor IX	F9
P01857	Immunoglobulin heavy constant gamma 1	IGHG1
P08473	Neprilysin	MME
P19256	Lymphocyte function-associated antigen 3	CD58
Q8N4U5	T-complex protein 11-like protein 2	TCP11L2
Q8N7R7	Cyclin-Y-like protein 1	CCNYL1
Q9NPF0	CD320 antigen	CD320
Q9Y4P8	WD repeat domain phosphoinositide-interacting protein 2	WIPI2
Q9Y689	ADP-ribosylation factor-like protein 5A	ARL5A
A4FU69	EF-hand calcium-binding domain-containing protein 5	EFCAB5
O00423	Echinoderm microtubule-associated protein-like 1	EML1
O95214	Leptin receptor overlapping transcript-like 1	LEPROTL1
P51530	DNA replication ATP-dependent helicase/nuclease DNA2	DNA2
Q14135	Transcription cofactor vestigial-like protein 4	VGLL4
Q32NC0	UPF0711 protein C18orf21	C18orf21
Q70CQ4	Ubiquitin carboxyl-terminal hydrolase 31	USP31
Q9H063	Repressor of RNA polymerase III transcription MAF1 homolog	MAF1
