# Supplementary material for: Downregulation of Brf1 Induces Liver Failure and Inhibits Hepatocellular Carcinoma Progression by Promoting Apoptosis
Source: J Cancer. 2024 Sep 3;15(17):5577–93. doi: 10.7150/jca.97277 (PMC11414613; doi:10.7150/jca.97277)
Supplement: Supplementary file 1 — Supplementary figures. [file jcav15p5577s1.pdf]

S1

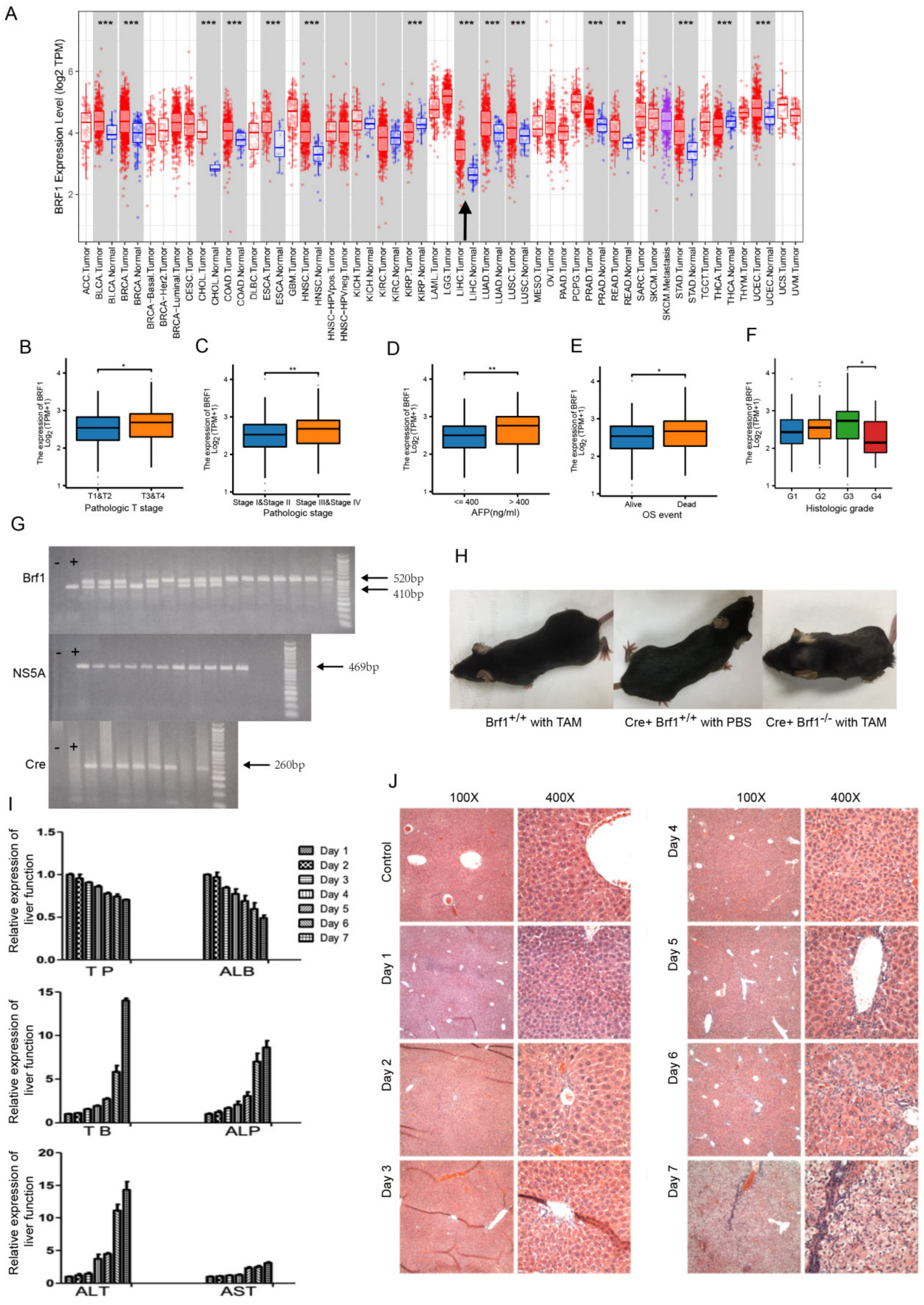

Supplementary Figure 1. Comprehensive Analysis of Brf1 Expression and Its Impact on Tumor Characteristics and Mouse Phenotype. (S1.A) Brf1 RNA Differential Expression in Tumor Patients. A boxplot illustrates the significantly higher

Brf1 expression in tumor tissues compared to normal tissues, as per the Human Protein Atlas (HPA) data (t test, ***p < 0.0001). (S1.B-F) Prognostic Value of Brf1 Expression. Brf1's prognostic significance is evident in various clinical parameters:

T-stage differentiation (T1+T2 vs. T3+T4 groups, t test, *p = 0.0112 < 0.05), Pathologic stage grouping (stage I + II vs. stage III + IV, t test, **p = 0.0057 < 0.01),

Alpha-fetoprotein (AFP) levels with normal distribution and even variance (t test, **p = 0.0033 < 0.01), Overall survival (OS) event with normal distribution and uneven variance (Welch t' test, *p = 0.0207 < 0.05), Histologic grade assessed by Welch One-way ANOVA Test (*p = 0.0387 < 0.05). (S1.G) Genotyping Results. The presence of Brf1 mutation and wildtype alleles is distinguished by their sizes: Brf1 mutant at 520bp, Brf1 wildtype at 410bp; NS5A at 469bp; Cre at 260bp. (S1.H) General Performance of Brf1 Knockout Mice. Phenotypic changes in Brf1 knockout mice include disheveled fur, hunched posture, lethargic movement, and tightened orbits. (S1.I) Time Course of Liver Function Changes. A time curve is established to monitor the gradual alterations in liver function without a distinct critical point. (S1.J) Time Course of Hepatocyte Pathological Changes. HE staining showed that the progression of hepatocyte damage is depicted as a gradual process without a sudden failure point.

S2

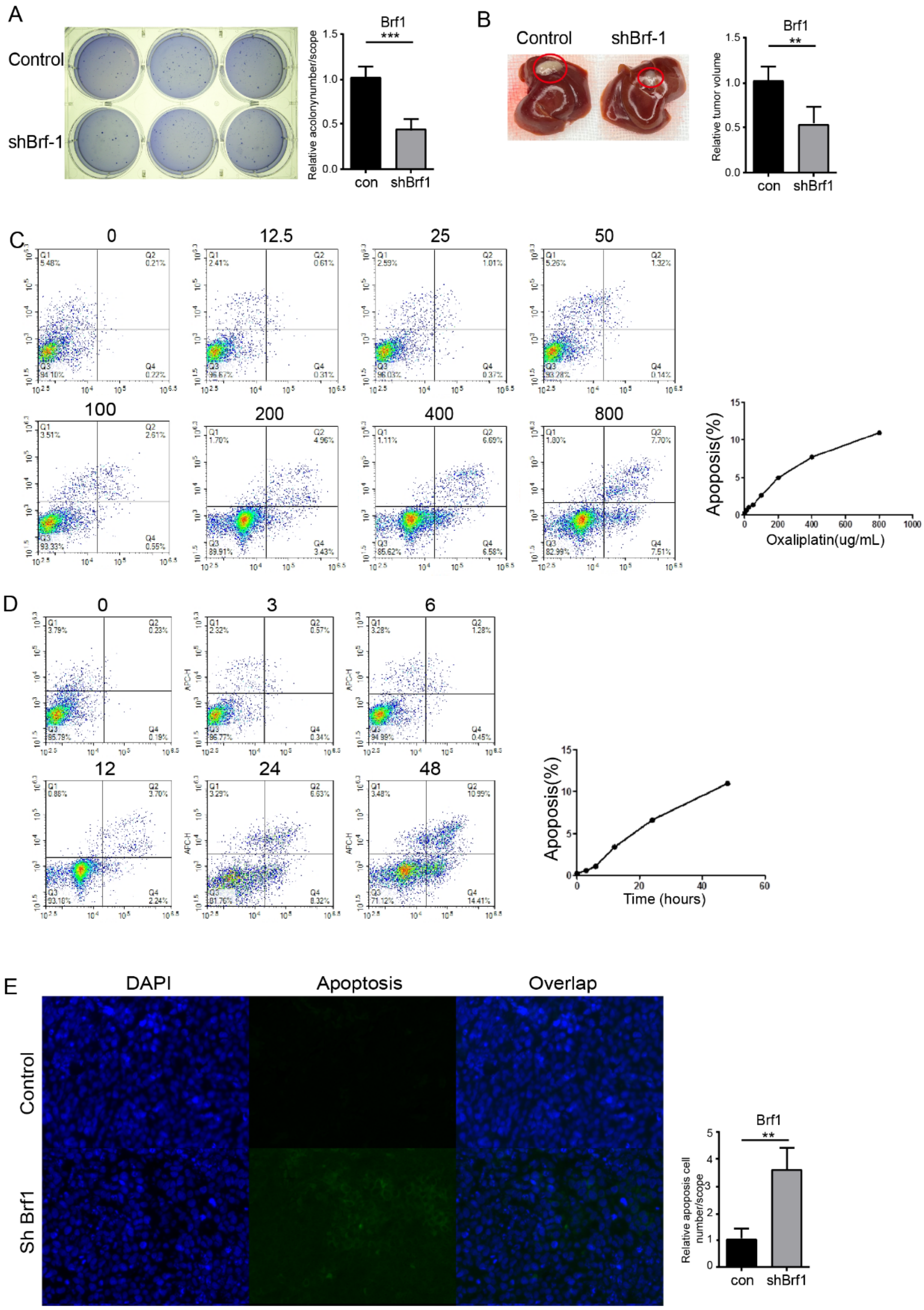

Supplementary Figure 2. Effects of Brf1 Knockdown on Colony Formation, Tumor Growth, and Chemotherapy-Induced Apoptosis. (S2.A) HepG2 Colony Growth in Agarose. Colonies of HepG2 cells exhibited a significantly slower growth rate in the Brf1 knockdown group compared to the control, as evidenced by the T test

results (***p < 0.001). (S2.B) HuH7 Subcutaneous Tumor Growth. The Brf1 knockdown group displayed a reduced rate of subcutaneous tumor growth when compared to the control group, with statistical significance indicated by the T test (**p < 0.01). (S2.C) Chemotherapy Drug Dosage Curve. Wild-type HepG2 cells treated with varying doses of oxaliplatin showed an increase in the apoptosis rate, which was measured by flow cytometry. The proportion of apoptotic cells increased progressively with higher concentrations of the chemotherapy drug. (S2.D) Chemotherapy Drug Time Course Curve. Wild-type HepG2 cells were treated with oxaliplatin for different durations, and the apoptosis rate was assessed using flow cytometry. The rate of apoptosis was observed to increase with longer exposure times to the drug. (S2.E) Tumor Tissue Cell Apoptosis Detected by TUNEL. Subcutaneous tumors from the Brf1 knockdown group demonstrated increased sensitivity to oxaliplatin-induced apoptosis at ×400 magnifications. The T test revealed a statistically significant difference in apoptosis rates between the Brf1 knockdown and control groups (**p < 0.001).

S3

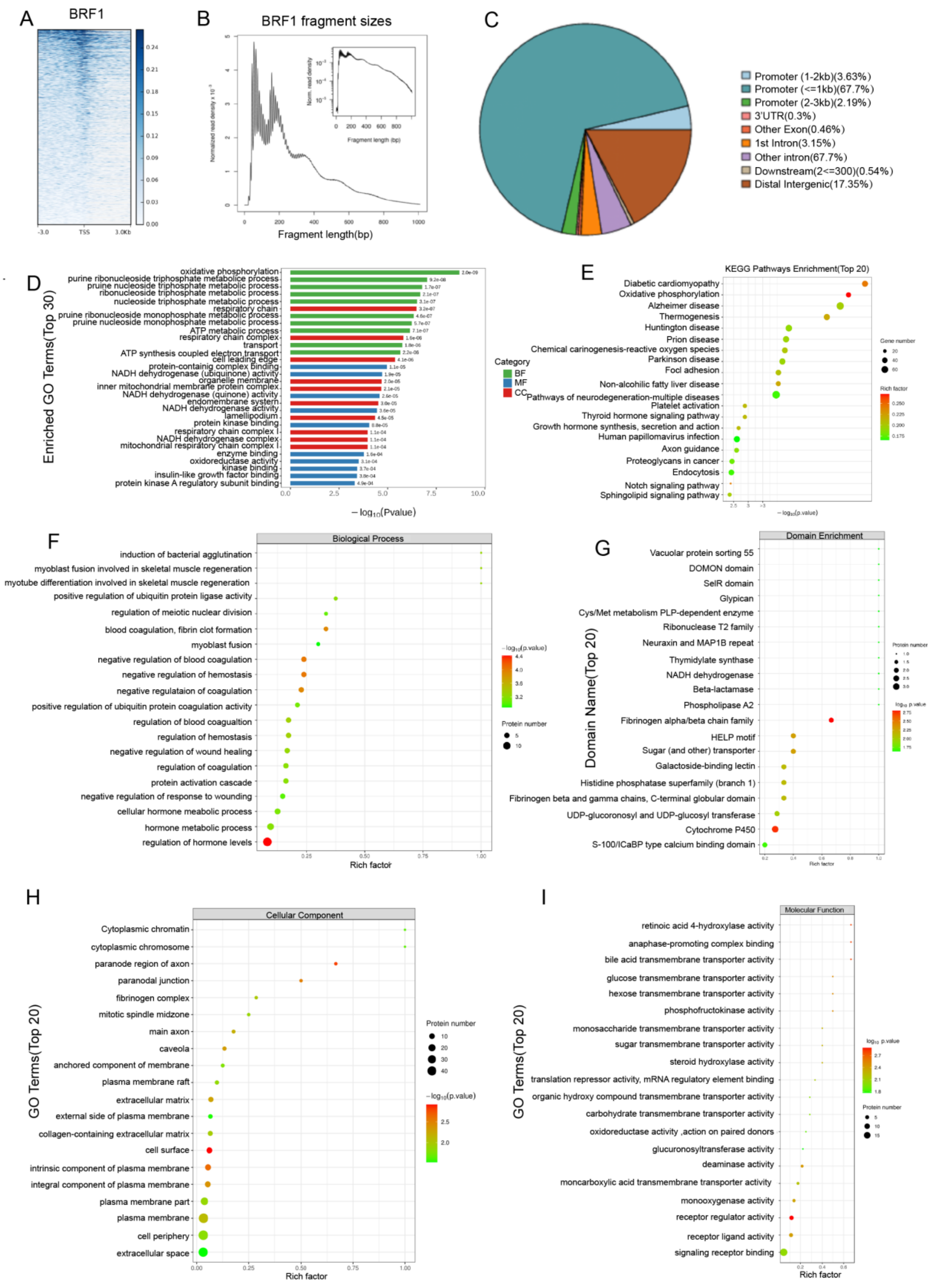

Supplementary Figure 3. Multi-Omics Analysis Revealing Brf1's Role in Gene Regulation, Metabolism, and Cellular Functions. (S3.A) Sequencing-Reads Distribution Relative to Transcription Start Site (TSS). The average signal intensity is depicted for regions 3kb upstream and downstream of the TSS, highlighting a

significant enhancement around the TSS. Notably, the upstream signal is stronger than the downstream, with a peak enhancement around 300 bp upstream, coinciding with the Brf1 binding site. Peaks are predominantly observed within 1000 bp, particularly concentrated 250 bp upstream and downstream of the TSS. (S3.B) Sequencing-Fragment Sizes Prediction. For a specific binding site, a significant enrichment of reads is observed, with the inserted fragment length ranging from 0-250 bp and distinct peaks at approximately 75 bp and 125 bp. (S3.C) Sequencing-Peak Distribution in Gene Functional Regions. This map confirms Brf1's role as a promoter for certain genes or gene classes. (S3.D) Transcriptomic-GO Enrichment Histogram (General). The genes are primarily associated with nucleic acid and energy metabolism, including processes related to purine ribonucleoside triphosphate, respiratory chain components, and ATP synthesis. (S3.E) Transcriptomic-KEGG Enrichment Bubble Diagram (General). The genes are linked to pathways involved in energy metabolism and chronic disease development, such as oxidative photosynthesis, thermogenesis, thyroid hormone signaling, and various diseases like diabetes, Alzheimer's, and Parkinson's. (S3.F) Proteomic-Domain Enrichment Analysis. Changes in Cytochrome P450 and sugar transporters suggest Brf1's significant impact on cellular energy metabolism, mainly located in mitochondria and the endoplasmic reticulum. Changes in fibrinogen alpha/beta chains indicate Brf1's role in blood coagulation. (S3.G) Proteomic-GO Functional Enrichment Bubble Diagram (BP). Brf1 is involved in the regulation of hormone levels, including thyroid hormone and growth hormone, which are integral to the body's energy metabolism, as well as various coagulation functions. (S3.H) Proteomic-GO Functional Enrichment Bubble Diagram (CC). Brf1's close association with the cell surface and plasma membrane components suggests its role in extrinsic and intrinsic apoptosis pathways. (S3.I) Proteomic-GO Functional Enrichment Bubble Diagram (MF). Brf1's relation to receptor regulator activity indicates its function through protein-protein interactions. The correlation with activities like bile acid and

glucose transmission, steroid hydroxylase, and carbohydrate transmission underscores Brf1's connection to liver metabolic functions.
